# Conformational Behavior and Self-Assembly of Gallol-Containing Block Copolymers in Non-Polar Media

**DOI:** 10.3390/molecules31152695

**Published:** 2026-08-03

**Authors:** Nikolay K. Balabaev, Puja Poddar, Subhadeep Shit, Sergei N. Chvalun, Priyadarsi De, Elena Yu. Kramarenko

**Affiliations:** 1 Enikolopov Institute of Synthetic Polymeric Materials RAS, Moscow 119991, Russia; balabaevnk@gmail.com (N.K.B.); s-chvalun@yandex.ru (S.N.C.); 2 Institute of Mathematical Problems of Biology RAS—The Branch of Keldysh Institute of Applied Mathematics of Russian Academy of Sciences, Pushchino 142290, Moscow Region, Russia; 3 Polymer Research Centre and Centre for Advanced Functional Materials, Department of Chemical Sciences, Indian Institute of Science Education and Research Kolkata, Mohanpur 741246, West Bengal, India; puja09.nab@iiserkol.ac.in (P.P.); ss23rs084@iiserkol.ac.in (S.S.); 4 Faculty of Physics, Lomonosov Moscow State University, Moscow 119991, Russia

**Keywords:** gallol-containing block copolymers, RAFT polymerization, molecular dynamics (MD) simulations, hydrogen bonding, self-assembly

## Abstract

While previous studies have illuminated the behavior of poly(gallol methacrylate)-*block*-poly(*N*-phenylmethacrylamide) copolymers (**PM-*b*-PNP**) in polar alcoholic mixtures, their performance in non-polar environments remains an uncharted territory. In this study, full-atomistic molecular dynamics (MD) simulations were first employed to investigate the conformational behavior and aggregation of experimentally relevant homo- and block copolymer architectures in heptane. The theoretical findings were subsequently supported through experimental studies using polymers synthesized via controlled reversible addition fragmentation chain transfer (RAFT) polymerization. Our results highlight the critical role of intramolecular hydrogen bonding in stabilizing specific conformations and helix-like structures of **PNP** homopolymers and the leading role of attractive van der Waals interactions in the aggregation of block copolymers.

## 1. Introduction

**Figure 1:**
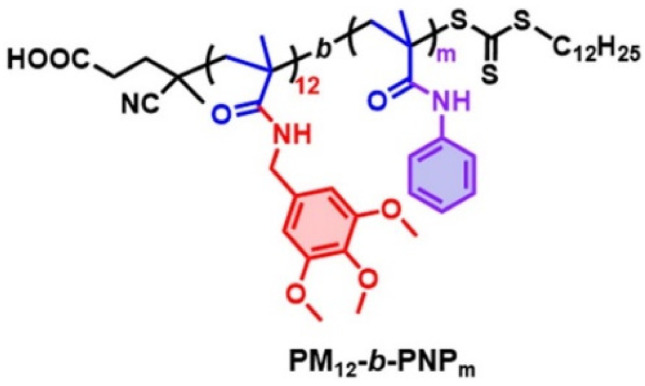
Chemical structure of the poly(gallol methacrylate)-*block*-poly(*N*-phenylmethacrylamide) copolymer (**PM_12_-*b*-PNP_m_**).

The rational design of functional polymeric materials relies fundamentally on a deep understanding of the relationship between molecular architecture, conformational behavior, and self-assembly mechanisms [1,2,3,4]. While synthetic strategies such as reversible addition fragmentation chain transfer (RAFT) dispersion polymerization have revolutionized the production of complex nano-architectures [5,6,7,8,9], the predictive power of these methods is ultimately grounded in the physics governing how individual polymer chains fold and organize in specific solvent environments. In our previous work [10], we successfully developed an approach to prepare an array of hierarchical antioxidant polymeric nano-architectures using gallol-functionalized stabilizers. We investigated the morphological evolution of poly(gallol methacrylate)-*block*-poly(*N*-phenylmethacrylamide) copolymers (**PM-*b*-PNP**) in polar alcoholic solvents, where the gallol-containing block acted as an intramolecularly amphiphilic solvophilic stabilizer (the chemical structure of **PM_12_-*b*-PNP_m_** block copolymer studied in [10] is shown in Figure 1). These materials belong to a new class of bioinspired, functional nano-architectures that offer significant potential for diverse applications, ranging from advanced protective coatings and targeted biomedical delivery systems to platforms designed for oxidative stress mitigation [11,12,13,14,15,16]. Through a combination of synthesis, experimental investigations, and coarse-grained modeling, we demonstrated that fine-tuning the degree of polymerization of the **PNP** block could drive precise morphology transitions—from micelles to worms and vesicles—by altering the delicate balance between solvophobic and solvophilic interactions. That study provided critical theoretical validation for the role of structural heterogeneity in defining equilibrium morphologies in alcoholic media.

However, the conformational landscape of these sophisticated macromolecules shifts dramatically when the solvent environment changes from polar to non-polar, a regime that remains largely unexplored yet holds significant implications for applications in hydrophobic drug delivery. In this work, we extend our investigation of the same **PM-*b*-PNP** block copolymer series, previously characterized in polar mixtures, into a non-polar solvent: heptane. This transition is not merely a change in medium but represents a fundamental shift in the physical identity of the polymer components. In heptane, the gallol-containing blocks, along with the *N*-phenylmethacrylamide (**NP**) segments of the second block, behave as strongly dipole entities. The high density of oxygen atoms on the gallol moiety and the polar amide groups in both the **PM** and **PNP** blocks generate substantial permanent dipole moments. In a low-dielectric medium where electrostatic screening is minimal, these dipoles engage in intense long-range interactions that dominate the conformational behavior. Consequently, the hydrogen bonds that form within and between chains can be interpreted as the local manifestation of strong dipole–dipole interactions, driving the system toward compact, energy-minimizing states distinct from those observed in polar solvents.

A pivotal factor influencing this new conformational behavior is expected to be the solubility of the polymer’s main chain relative to its polar pendant groups. In our previous studies in alcoholic solvents [7], the behavior was governed by the classic amphiphilic balance where the gallol block was solvophilic. In heptane, however, the scenario is inverted and more complex: the hydrocarbon backbone is well-solvated and seeks to expand, while both the gallol side chains and the **NP**-containing blocks possess significant polarity that drives them to collapse or associate to minimize exposure to the non-polar environment. This creates a unique tension where the chemical nature of the main chain acts as a structural scaffold that may resist the aggregation driven by the dipolar side chains and the second block. Investigating this balance is crucial for deciphering the self-organization mechanisms of these graft-like polymers. Key questions arise: Does the solvated backbone force the chain into an extended coil despite strong dipolar attractions? Or do the collective dipolar interactions of both the gallol and **NP** blocks overcome the entropic penalty to form dense, kinetically trapped globules? Furthermore, how do these single-chain conformations evolve into multi-chain aggregates when the solvophilic/solvophobic definition is fundamentally altered?

To address these fundamental questions, this work presents a comprehensive computational investigation of the conformational behaviour and complexation of **PM-*b*-PNP** block copolymers, as well as their constituent homopolymer blocks, in heptane using full-atomistic molecular dynamics (MD) simulations. The experimentally synthesized and structurally characterized polymers provide chemically realistic model systems for the atomistic simulations, enabling direct correlation between polymer architecture and aggregation behavior in non-polar media. Unlike coarse-grained (CG) approaches which were sufficient to capture large-scale morphological transitions in polar media, the specific nature of dipole interactions and hydrogen bonding networks in non-polar solvents requires the high chemical fidelity of atomistic modeling. Our methodology resolves the intricate network of intramolecular and intermolecular hydrogen bonds and explicit dipole interactions within single chains and small oligomers with precision. This allows for a mechanistic understanding of how specific chemical groups dictate local folding, stability, and the formation of helix-like or globular structures. We acknowledge that this choice limits the accessible timescales and system sizes compared to CG methods, representing a trade-off between chemical fidelity and scale that is appropriate for the mechanistic questions addressed here. By systematically analyzing systems with individual blocks and block copolymers, we aim to elucidate the specific influence of backbone solubility and the dipole strength of both the gallol and **PNP** blocks on the overall architecture. This study not only continues our exploration of these bioinspired nanomaterials by mapping their behaviour across different solvent polarities but also provides rigorous atomic-level insights into the physics of dipolar polymers, offering a theoretical foundation for predicting their self-assembly in non-aqueous applications. Beyond the specific chemical system investigated, this work contributes to the fundamental understanding of dipolar polymers [17,18,19,20,21,22,23,24,25,26] and graft architectures [27,28,29,30,31,32]. The conformational behavior of chains bearing strong permanent dipoles in low-dielectric media remains a critical challenge in soft matter physics, where electrostatic interactions often compete with entropic elasticity and excluded volume interactions [26]. Furthermore, the dense arrangement of gallol side groups presents a model system for graft-like polymers, where steric constraints and side-chain interactions dictate the overall chain stiffness and solvation properties. By elucidating the interplay between backbone solubility and dipolar side-chain association, our study provides generalizable insights that extend to a broader class of functionalized macromolecules, bridging the gap between specific chemical design and universal physical principles governing polymer self-assembly.

## 2. Results

Our atomistic MD study proceeds in two stages. First, we examine the conformational behavior of individual homopolymer chains (**PM_50_** and **PNP_50_**) in heptane. Second, we extend the analysis to **PM_10_-*b*-PNP_40_** block copolymers, simulating systems containing one, two, and four chains to probe concentration-dependent effects from the isolated-chain limit up to semi-dilute conditions (25 wt%).

### 2.1. Single-Chain Conformations

#### 2.1.1. Structure and Hydrogen Bonding of the **PNP_50_** Polymer

**Figure 2:**
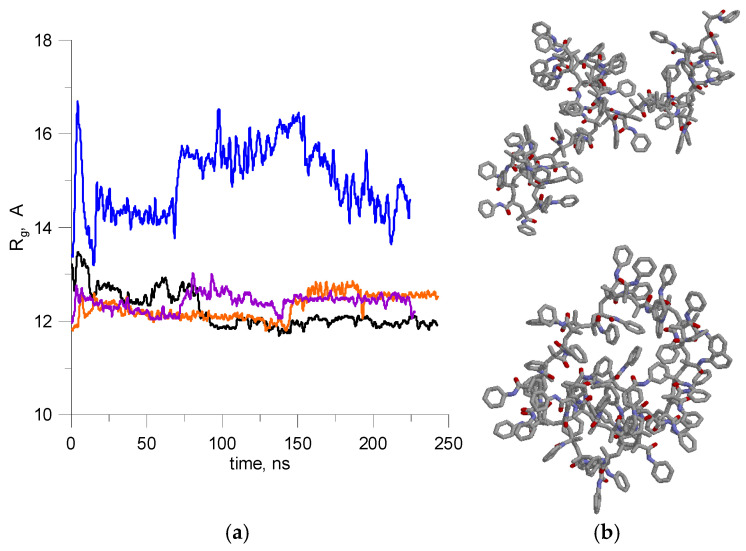
(**a**) Time dependence of the radius of gyration *R*_g_ for **PNP_50_** in heptane at 300 K. The curves correspond to the four independent simulation variants shown by different colors. (**b**) Typical final structures of the **PNP_50_** polymer for the simulation trajectories shown by blue (upper conformation) and orange (bottom conformation) colors in (**a**). The polymers are displayed as skeletal models in their maximum area projection; hydrogen atoms are omitted for clarity.

In the case of the **PNP_50_** polymer, the structural evolution presents a striking example of stability driven by internal attractive forces both at 300 and 350 K. Time dependences of the radius of gyration presented in Figure 2a, which remain relatively constant, indicating a lack of significant expansion or unfolding, demonstrate that the polymer retains a compact, globular conformation throughout the simulation duration at both temperatures. The only notable deviation occurs in one specific run, where the chain exhibits a slightly more expanded conformation and increased fluctuation amplitude, suggesting that the chain is rather in a coil state subjected to thermal fluctuations. This conclusion is confirmed by visual inspection of the skeletal models, stripped of hydrogen atoms for clarity and oriented along their maximum area projection as shown in Figure 2b.

The primary mechanism responsible for this remarkable stability of different conformations is the formation of an extensive network of intramolecular hydrogen bonds, specifically of the O⋯H-N type, formed between the carbonyl oxygens and the amide protons of the polymer. Although theoretical models might predict the presence of N⋯H-N interactions between different monomers, our analysis confirms that such bonds are negligible in number and impact, appearing only sporadically between adjacent units without influencing the global architecture.

**Figure 3:**
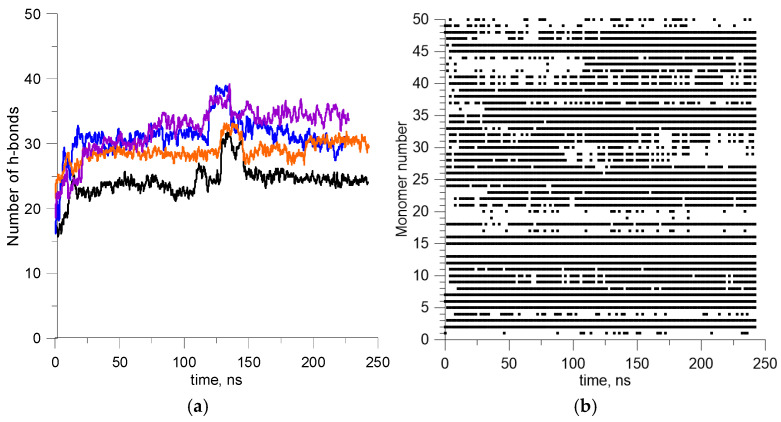
(**a**) Time evolution of the total number of intramolecular hydrogen bonds in **PNP_50_** for the four simulation variants. The color coding matches the curves in Figure 2a. (**b**) A typical map illustrating the participation of individual monomers (residues 1–50) in hydrogen bonding over time (sampled every 2 ns) for **PNP_50_** at 300 K. Dots indicate the presence of a hydrogen bond involving a specific monomer at a given time.

**Figure 4:**
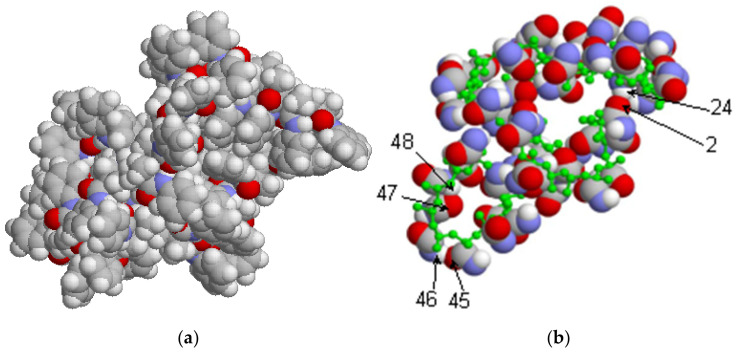
(**a**) Volumetric representation of the **PNP_50_** molecule at *t* = 200 ns and (**b**) a detailed view of the atoms involved in hydrogen bonding. The backbone is shown as a green ball-and-stick model (hydrogens omitted), while carbonyl (CO) and amide (NH) groups are highlighted. Specific long-range hydrogen bonds (e.g., between monomers 2 and 24) are labeled, demonstrating how distant chain segments are bridged to stabilize the globule.

**Figure 5:**
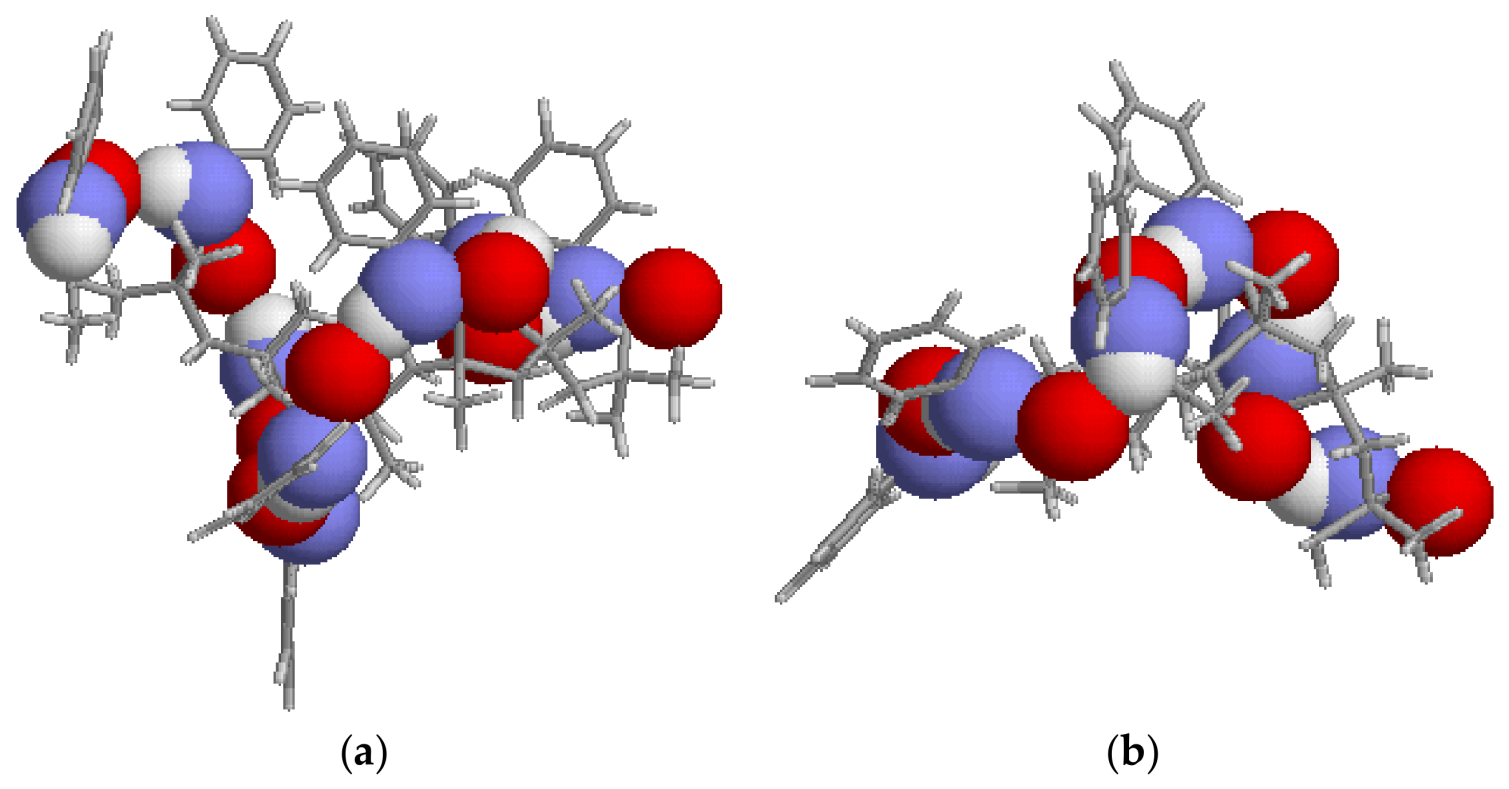
Examples of stable helix-like segments formed within the **PNP_50_** chain: (**a**) an 8-monomer segment (residues 3–10) and (**b**) a 6-monomer segment (residues 45–50). Oxygen and amide atoms are rendered as space-filling models, while the rest of the chain is skeletal. These locally ordered structures persist for tens of nanoseconds, adding rigidity to the overall conformation.

A detailed quantitative analysis of the hydrogen bonding landscape in **PNP_50_** reveals that the number of these stabilizing interactions remains remarkably steady, averaging between 25 and 31 bonds depending on the specific simulation trajectory, as illustrated in Figure 3a. This high occupancy implies that the vast majority of monomeric units are engaged in hydrogen bonding at any given instant. Temporal mapping of bond participation, depicted in Figure 3b for one particular variant (bond participation maps calculated for other variants and also for elevated temperature of 350 K are similar), further illustrates that most monomers maintain these connections continuously over hundreds of nanoseconds, with only minor fluctuations. While the dominant contribution comes from bonds between adjacent monomers along the chain, the structural integrity of the globule is significantly reinforced by specific long-range interactions that act as molecular “staples.” Such long-range contacts exhibited exceptional longevity, remaining intact for over 90% of the analyzed frames and reforming almost immediately upon transient breakage. The volumetric model in Figure 4 provides a clear visualization of these critical contacts, highlighting how distant parts of the chain are locked together. Furthermore, localized regions of the chain occasionally organize into helix-like segments, where consecutive hydrogen bonds lock several monomers into a rigid, spiral arrangement as shown in Figure 5, contributing further to the overall compactness and reduced mobility of the **PNP_50_** globule.

#### 2.1.2. Conformational Behavior and Hydrogen Bonding of the **PM_50_** Polymer

**Figure 6:**
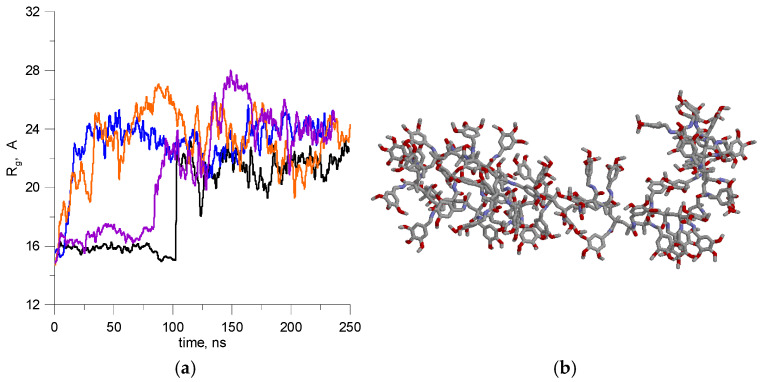
(**a**) Time dependence of the radius of gyration *R*_g_ for **PM_50_** in heptane across four independent variants at 300 K shown by different colors. The plots reveal diverse unfolding pathways, ranging from gradual expansion to sharp, step-like transitions, reflecting the complex energy landscape of the **PM_50_** chain. (**b**) An example of a final (*t* = 240 ns) structure of the **PM_50_** polymer is displayed as skeletal models. Hydrogen atoms are omitted.

**Figure 7:**
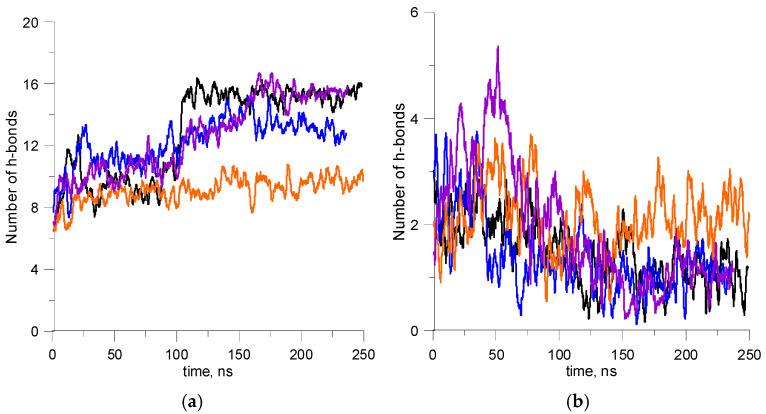
Time evolution of the number of intramolecular hydrogen bonds in **PM_50_,** separated by type: (**a**) backbone O⋯H-N bonds and (**b**) side-chain Os⋯H-N bonds. The curves represent running averages over 1 ns (the curves for four variants are shown by different colors). While backbone bonds are numerous (10 ÷ 16) and relatively stable, side-chain bonds are sparse (1 ÷ 2) and highly fluctuating, indicating their transient nature.

**Figure 8:**
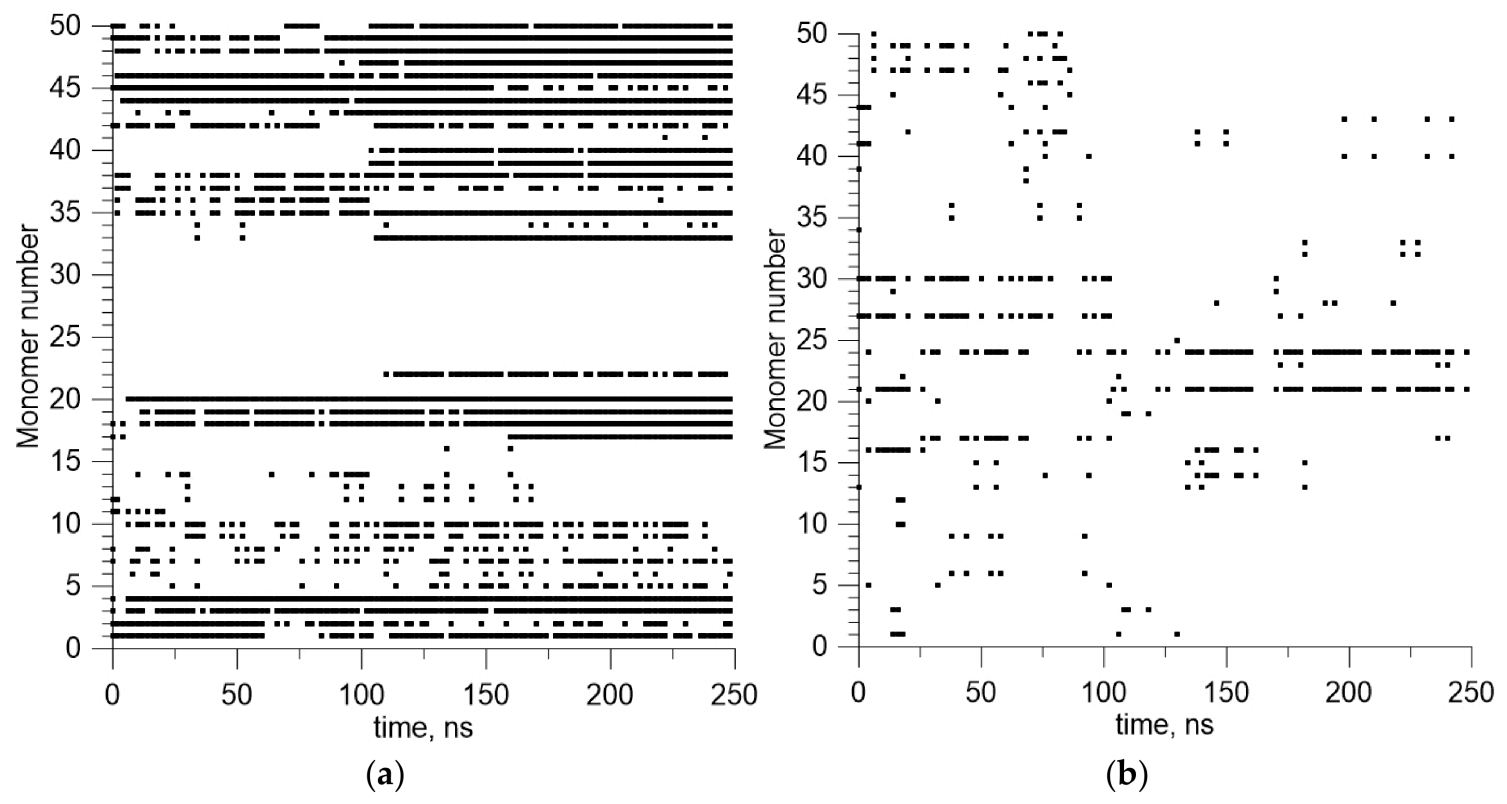
Maps showing the participation of individual monomers in hydrogen bonding for **PM_50_**, sampled every 2 ns. Panel (**a**) displays backbone O⋯H-N bonds, revealing that some monomers are consistently bonded while others are intermittent. Panel (**b**) displays side-chain Os⋯H-N bonds, which appear as sparse, flickering dots, except for a distinct persistent trace corresponding to the stable 21–24 interaction.

In stark contrast to the static nature of **PNP_50_**, the **PM_50_** polymer displays a highly dynamic conformational behavior characterized by a transition from a compact globule to an expanded coil. This unfolding process is evident from time dependence of the radius of gyration that shows a clear trend toward larger values over time, as plotted in Figure 6 and in visual snapshots (an example is shown in Figure 6b). The transition mechanisms vary between simulation runs, manifesting either as a smooth, gradual expansion or as abrupt, cooperative jumps in dimensions, indicative of sudden structural rearrangements. This behavioral difference stems from the unique chemical architecture of **PM_50_**, which incorporates gallol side groups containing ether oxygens capable of participating in hydrogen bonding. Consequently, the **PM_50_** system supports three distinct types of potential hydrogen bonds: the backbone O⋯H-N interactions, side-chain mediated Os⋯H-N bonds involving the ether oxygens, and the negligible N⋯H-N contacts. Statistical analysis reveals that while the backbone O⋯H-N bonds remain the most abundant, averaging 10 to 16 per frame as shown in Figure 7a and Figure 8a, the involvement of side chains introduces a layer of complexity and variability absent in **PNP_50_**. The Os⋯H-N interactions are significantly less frequent, typically numbering only one or two at any instant as seen in Figure 7b and Figure 8b, and generally exhibit a transient, “blinking” character.

**Figure 9:**
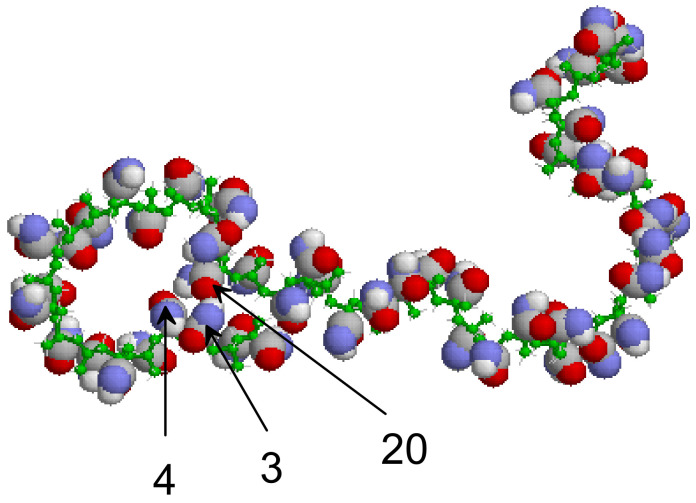
Detailed view of the **PM_50_** backbone atoms involved in long-range hydrogen bonds at *t* = 240 ns. The image highlights specific pairs (monomers 3–20 and 4–20) that form stable bridges between distant parts of the chain. These interactions act as topological constraints, influencing the rate and pathway of the polymer’s unfolding.

Despite the generally fleeting nature of side-chain interactions in **PM_50_**, specific instances of long-lived stability were observed, highlighting the potential for these groups to act as conformational locks under certain conditions. In one notable case, a hydrogen bond formed between the amide group of monomers and the ether oxygen of monomer persisted for over 130 nanoseconds, demonstrating that side-chain interactions can occasionally dominate the local dynamics. The h-bond maps in Figure 8 provide a comprehensive view of this temporal behavior, distinguishing between the persistent backbone bonds and the intermittent side-chain contacts. Similar to **PNP_50_**, the backbone hydrogen bonding in **PM_50_** is primarily local, occurring between neighboring monomers, yet long-range bridges also play a critical role in modulating the unfolding pathway. Several persistent long-range contacts were identified, which formed early in the simulation and remained stable until the end. These enduring links suggest that even as the overall chain expands, specific topological constraints imposed by distant interactions can temporarily trap the polymer in intermediate states, slowing down the relaxation process. Figure 9 visualizes these critical contacts, emphasizing how specific atomic arrangements can create kinetic traps within the rugged energy landscape of **PM_50_**, allowing for a rich variety of conformational states that are sensitive to both thermal history and specific atomic arrangements.

The conformational behavior of the polymers investigated in this study is fundamentally governed by the presence of chemical groups featuring separated charges, effectively creating intrinsic electric dipoles along the chain. In this context, the extensive network of intramolecular hydrogen bonds observed in our atomistic simulations can be physically interpreted as the direct result of favorable dipole–dipole interactions, where the alignment of these local dipoles minimizes the system’s electrostatic energy. This perspective aligns closely with previous coarse-grained studies [26,33,34,35], which demonstrated that the conformational landscape of dipolar chains is dictated by a delicate balance between electrostatic forces, which are strongly modulated by solvent polarity, chain stiffness, and van der Waals interactions. Those works highlighted that dipolar segments have a natural tendency to self-organize into linear aggregates to maximize electrostatic gain; in our specific systems, this driving force manifests structurally as the formation of stable, helix-like segments where consecutive monomers align their dipoles in a cooperative spiral arrangement. Furthermore, the earlier research [26] established that for shorter chains, the energy barrier between different compact states is often comparable to thermal energy, allowing the polymer to access multiple metastable conformations and undergo transitions between them driven by thermal fluctuations. This phenomenon provides a robust theoretical framework for understanding the behavior of our **PM_50_** polymer, which samples various expanded and collapsed states, while also explaining the exceptional stability of the **PNP_50_** globule, where the sheer density of dipolar interactions (hydrogen bonds) creates a deep energy well that suppresses such thermally activated transitions, locking the chain into a single, rigid equilibrium structure.

### 2.2. Aggregation of ***PM_10_*-b-*PNP_40_*** Block Copolymers in Heptane

To systematically investigate the early stages of supramolecular organization, we performed atomistic MD simulations of **PM_10_-*b*-PNP_40_** block copolymers at increasing concentrations: single-chains (15.4 wt%), two and four chains in the simulation box (25 wt% for both of the systems). Although the concentration 15.4 wt% for the single chain system is relatively high, the dimensions of the simulation cell were sufficient to prevent the polymer from interacting with its periodic images. Consequently, the system effectively models a polymer in a dilute solution regime, isolating intramolecular interactions from intermolecular aggregation. The intermolecular aggregation mechanisms are further elucidated from two and four block copolymers systems at enhanced concentration.

#### 2.2.1. Morphological Evolution and Percolation Behavior

**Figure 10:**
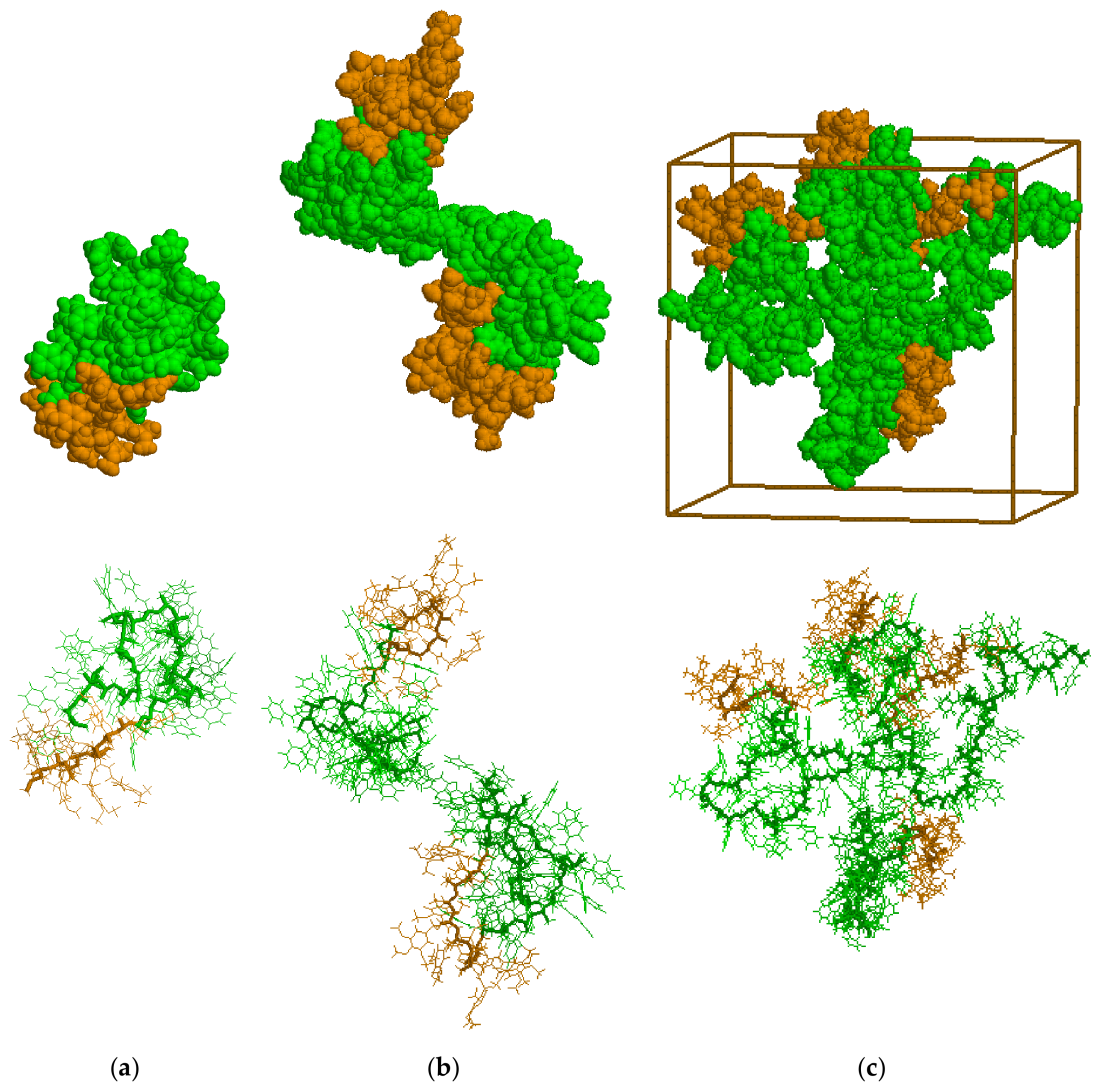
Comparative snapshots of **PM_10_-*b*-PNP_40_** systems at different concentrations: (**a**) single chain; (**b**) two chains at 25 wt%; (**c**) four chains at 25 wt%. The top row displays space-filling models, while the bottom row shows skeletal models with the backbone highlighted. The **PM_10_** blocks are colored orange, and the **PNP_40_** blocks are green.

Across all concentrations, the **PM_10_-*b*-PNP_40_** copolymers exhibit a consistent structural motif: extended, solvated gallol-containing **PM_10_** blocks coupled with collapsed, associative **PNP_40_** blocks. However, the manner in which these building blocks organize into larger assemblies depends critically on the number of block copolymer molecules in the simulation box. Figure 10 presents a unified comparison of representative snapshots from single-chain, two-chain, and four-chain systems after 100–120 ns of relaxation.

At the single-chain level, the copolymer adopts a compact globular conformation stabilized by intramolecular interactions, with no intermolecular contacts possible by definition. Upon doubling the number of chains at 25 wt%, the molecules begin to associate through their **PNP_40_** blocks, forming transient but persistent contacts that occasionally span the simulation box via periodic boundaries. In the four-chain system (also 25 wt%), this tendency culminates in the formation of stable percolating structures. All four independent variants of the four-chain system exhibit percolation along the vertical axis of the simulation box, with one variant additionally displaying horizontal connectivity, effectively forming a two-dimensional network.

Detailed inspection of individual molecules within the two- and four-molecule complexes (Figure 10) reveals that each chain retains its characteristic globular architecture, with the **PM_10_** blocks preferentially contacting the surface of **PNP_40_** domains rather than penetrating their core. This structural arrangement suggests that the gallol-containing blocks, while polar, remain partially exposed to the solvent even within aggregates, potentially preserving their functional accessibility for antioxidant or metal-chelating applications.

**Figure 11:**
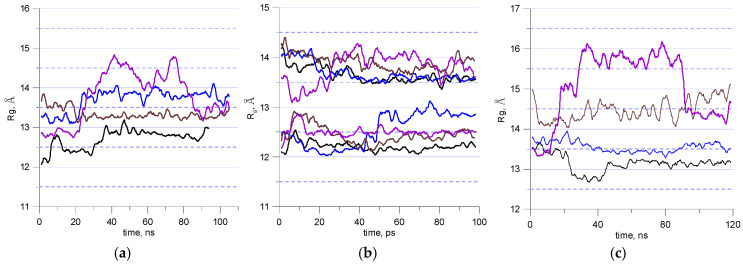
Time dependence of the radius of gyration for the **PM_10_-*b*-PNP_40_** copolymer (**a**) for single chain system, (**b**) for two-chain system at 25 wt%, and (**c**) four-chain system at 25 wt%. Data represents running averages over 2 ns. Different colors for single and two-chain systems panels (**a**,**b**) correspond to the four independent initial configurations. For panel (**c**) different colors correspond to different molecules for one of the variants of simulation runs.

The block copolymer size, characterized by the radius of gyration, for single-chain system as well as for systems with two and four molecules fluctuates with relatively small amplitudes around distinct mean values once the systems reach a stationary regime. The realized *R*_g_ values lay in the interval from roughly 12 A till 15 Å independent from the number of chains in the simulation box (Figure 11).

#### 2.2.2. Hydrogen Bonding Networks: Intra- vs. Intermolecular Contributions

The stability of both single-chain conformations and multi-chain aggregates is profoundly influenced by hydrogen bonding. Remarkably, the average number of intramolecular O⋯H-N bonds per chain remains relatively constant (31 ÷ 38 bonds) regardless of concentration, indicating that the internal folding of each copolymer is primarily governed by local chemical preferences rather than intermolecular crowding.

**Figure 12:**
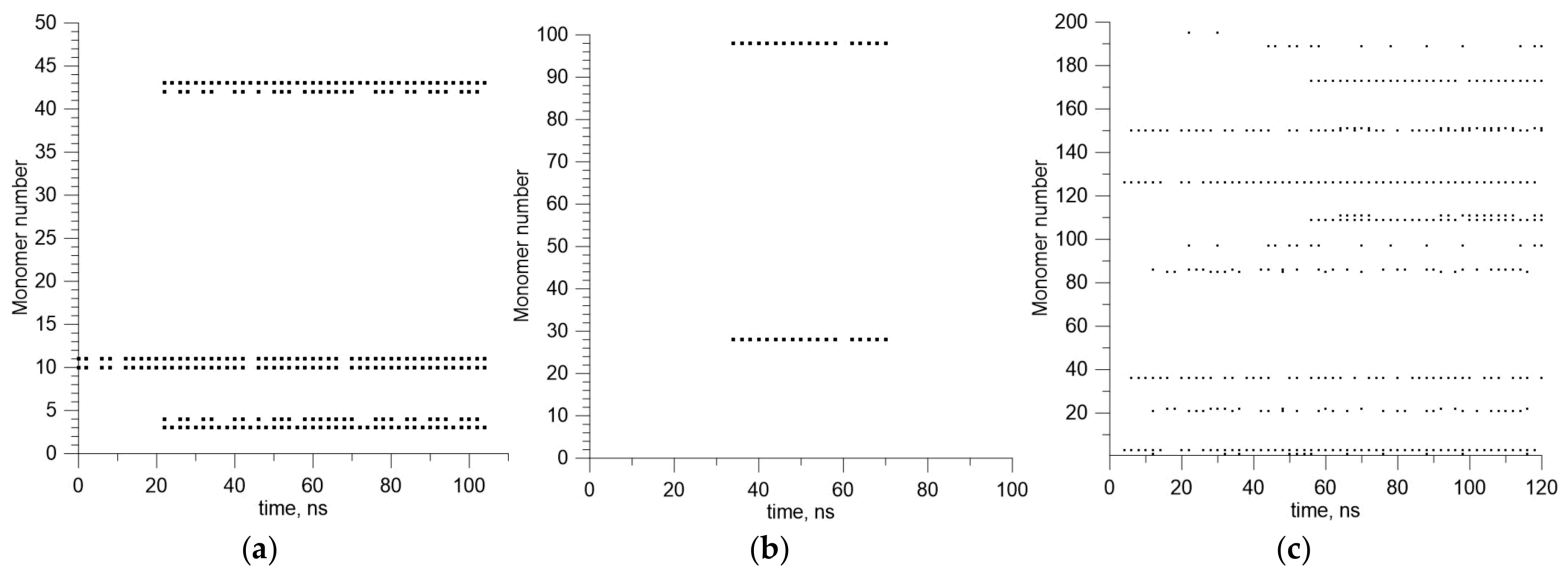
Maps illustrating (**a**) monomer participation in hydrogen bonding with a 2 ns sampling interval specifically for hydrogen bonds formed between the **PM_10_** and **PNP_40_** blocks in single-chain system; (**b**,**c**) chain participation in intermolecular hydrogen bonding in two (**b**) and four (**c**) **PM_10_-*b*-PNP_40_** copolymer systems.

The vast majority of these bonds occur between adjacent monomers, providing local rigidity to the chain. However, a critical subset of 2 ÷ 4 long-range hydrogen bonds per chain, which are formed between distant segments along the contour, acts as conformational “staples” that lock the global architecture. These long-range contacts emerge stochastically during the relaxation process and, once formed, exhibit exceptional persistence, often lasting for the remainder of the trajectory. Thus, while the general globular morphology is conserved, the specific internal topology is unique to each trajectory, defined by the stochastic formation of these critical long-range links. It should be noted that the amount of interblock hydrogen bonds is also very small (2 ÷ 3 bonds between **PM** and **PNP** blocks in single chain system, Figure 12a).

In contrast to the robust intramolecular network, intermolecular hydrogen bonds are notably scarce. Figure 12b,c illustrate that in the dimer and tetramer systems, only 2 ÷ 4 intermolecular H-bonds exist at any given time, connecting specific pairs of chains. While quantitatively minor, these sporadic contacts may play a disproportionate role in nucleating and stabilizing the early stages of aggregation by providing directional “guidance” for chain association. The predominance of van der Waals interactions over hydrogen bonding in driving aggregation is further supported by contact analysis, as discussed below.

#### 2.2.3. Van Der Waals Contacts and the Mechanism of Aggregation

To quantify the non-specific interactions that drive aggregate formation, we analyzed van der Waals contacts between atoms of different chains, defining a contact as any interatomic distance below 4 Å. Table 1 presents the average number of van der Waals interchain contacts per chain in two- and four-chain systems.

For four-chain system, the analysis reveals a clear hierarchy: **PNP-PNP** contacts dominate the intermolecular interaction landscape, followed by **PM-PNP** contacts, while **PM-PM** contacts are virtually absent. This pattern is consistent across all four independent variants and aligns with the chemical intuition that the more polar **PNP_40_** blocks, being poorly solvated in heptane, seek to minimize their exposure to the non-polar solvent by associating with one another. The **PM_10_** blocks, while also polar, appear to act primarily as “linkers” that bridge **PNP_40_** domains rather than forming extensive homotypic contacts. The somewhat distinct distribution of contact numbers observed in the two-chain system appears to be associated with the specific architecture of the percolating cluster and reflects finite-size effects.

#### 2.2.4. Solvation Shell Analysis: Concentration-Dependent Desolvation

A complementary perspective on aggregation is provided by analyzing the solvation shells of the polymers. We define a heptane molecule as belonging to the solvation shell of a polymer if its center of mass lies within 4 Å of any polymer atom. Table 2 summarizes the average number of solvent molecules per polymer (and per monomer) across the three concentration regimes.

The data reveal a systematic decrease in solvation with increasing polymer concentration, reflecting the progressive burial of polymer surface area within aggregates. Notably, the **PM_10_** blocks retain a higher degree of solvation than **PNP_40_** blocks across all concentrations, consistent with their more extended, solvent-exposed conformation. The fact that the sum of solvation numbers for isolated **PM_10_** and **PNP_40_** blocks exceeds that of the intact copolymer indicates significant overlap in their solvation shells, i.e., some heptane molecules simultaneously solvate both blocks at the interface.

This concentration-dependent desolvation provides a thermodynamic driving force for aggregation: as polymer concentration increases, the system can lower its free energy by reducing the total polymer–solvent interfacial area through chain association. The preferential desolvation of **PNP_40_** blocks further explains why these domains serve as the primary “sticky” patches that nucleate aggregate formation.

Taken together, these results construct a coherent picture of concentration-dependent self-assembly in **PM_10_-*b*-PNP_40_**/heptane systems. At low concentration (single chain), intramolecular hydrogen bonding and dipole interactions dominate, yielding stable, compact globules with exposed **PM_10_** blocks. As concentration increases, the intermolecular association takes place, primarily through **PNP-PNP** contacts that minimize unfavorable polymer–solvent interfaces. The resulting aggregates retain the internal architecture of their constituent chains while establishing new intermolecular contacts that can span the simulation box via periodic boundaries.

The scarcity of intermolecular hydrogen bonds suggests that aggregation is driven primarily by non-specific van der Waals interactions and solvophobic effects rather than directional H-bonding. This has important implications for the dynamics of the system: aggregates are likely to be fluid and reconfigurable on experimental timescales, rather than kinetically trapped in rigid architectures. The persistent solvation of **PM_10_** blocks within aggregates further suggests that the functional gallol groups may remain accessible for interactions with external species in precipitate.

These atomistic insights provide a mechanistic foundation for interpreting experimental observations of precipitation and gelation in heptane.

### 2.3. Macroscopic Aggregation Behaviour of ***PM_10_*-b-*PNP_40_*** Block Copolymers in Heptane

**Figure 13:**
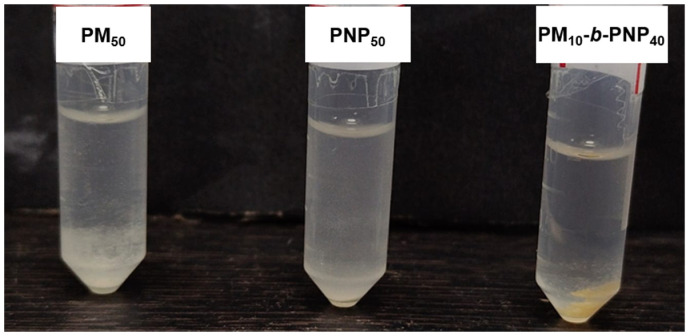
Visual observation of polymer aggregation in heptane.

To experimentally examine the aggregation behaviour of the synthesized polymers in a non-polar environment, the polymers were first dissolved in a minimum amount of THF and subsequently added dropwise into heptane under continuous stirring. The resulting dispersion was stirred for 5–6 h, followed by gradual evaporation of THF by keeping the Eppendorf tube cap open at room temperature for 24 h. Upon removal of THF, the polymers gradually formed visible precipitates in heptane, indicating poor solubility and strong intermolecular association in the non-polar medium (Figure 13). The precipitation behavior suggests that the highly dipolar gallol and **PNP** segments are thermodynamically incompatible with the non-polar solvent environment, promoting polymer–polymer interactions over polymer–solvent interactions.

The observed aggregation behaviour is consistent with the molecular dynamics simulation results, which revealed dominant intermolecular van der Waals interactions and hydrogen-bond-driven association among polymer chains in heptane. These interactions facilitate chain collapse and aggregation, ultimately leading to precipitation in the non-polar medium. Direct morphological characterization of the precipitated aggregates by small-angle X-ray scattering (SAXS), transmission electron microscopy (TEM), scanning electron microscopy (SEM), or atomic force microscopy (AFM) could provide complementary information on aggregate size and morphology. However, such analyses were beyond the scope of the present study.

## 3. Discussion

The comprehensive molecular dynamics study of **PM_50_** and **PNP_50_** polymers, alongside experimental observations and simulations of **PM_10_-*b*-PNP_40_** block copolymers in heptane, leads to several fundamental conclusions regarding the conformational behaviour and self-organization of these systems in non-polar media. The experimentally synthesized polymers and their precipitation behaviour in heptane provide important macroscopic validation for the aggregation tendencies and intermolecular interactions predicted by the atomistic simulations.

First, the conformational behavior of a polymer chain in a good solvent for its backbone is not dictated solely by solvent quality but is profoundly governed by the specific architecture of side chains and the resulting intramolecular hydrogen bonding networks. The **PNP_50_** polymer demonstrates exceptional structural stability, maintaining a compact globular state even at elevated temperatures (350 K). This rigidity is directly attributed to O⋯H-N hydrogen bonds, involving nearly every monomer unit. Crucially, the **PNP_50_** conformations are stabilized by long-range “staple” interactions between distant segments of the chain and the formation of transient helix-like motifs, which collectively create a high energy barrier against unfolding. In contrast, the **PM_50_** polymer, despite possessing a similar backbone, exhibits significant conformational plasticity, transitioning from a globule to an expanded coil. This difference arises from the presence of bulky gallol side groups, which introduce steric hindrance and compete for hydrogen bonding partners, thereby disrupting the continuity of the hydrogen bond aggregates and lowering the stability of the collapsed state.

Second, the nature and lifetime of hydrogen bonds play a decisive role in defining the dynamic landscape of these macromolecules. While backbone-mediated O⋯H-N bonds serve as the primary structural scaffold in both systems, their population and persistence differ markedly: **PNP_50_** maintains a high, constant count of ~25 ÷ 30 bonds, whereas **PM_50_** sustains a lower, fluctuating count of ~10 ÷ 16 bonds. Furthermore, the introduction of ether oxygens in the **PM_50_** side chains adds a layer of complexity through transient Os⋯H-N interactions. Although generally short-lived, these side-chain bonds can occasionally form remarkably stable, long-duration locks (persisting for over 100 ns), acting as kinetic traps that modulate the unfolding pathway. This suggests that side-chain chemistry does not merely act as a passive steric bulk but actively participates in the conformational kinetics, creating a rugged energy landscape characterized by multiple metastable states.

Third, the transition from single-chain behavior to multi-chain aggregation reveals the early stages of supramolecular organization driven by block incompatibility. In the case of **PM_10_-*b*-PNP_40_** block copolymers, the **PNP** blocks act as associative domains, driving the formation of dynamic, fluid clusters even in a dilute regime. The observation of emerging percolating structures indicates that these fleeting contacts are sufficient to initiate network formation. This implies that while individual aggregates lack long-term stability, the collective system possesses an inherent tendency toward gelation or phase separation, driven by the microphase separation of the solvophobic blocks. The atomistic insights gained here will inform the parameterization of system-specific coarse-grained models for studying larger-scale assembly in future work.

Finally, the ability to reproduce experimental observations—such as the precipitation of these polymers in heptane—through the careful analysis of hydrogen bonds and aggregate morphologies provides a robust mechanistic explanation for macroscopic behavior. These findings offer valuable guidelines for the rational design of graft-like block copolymers, suggesting that tuning the balance between backbone hydrogen bonding potential and side-chain steric/electronic properties allows for precise control over material stiffness, solubility, and self-assembly capabilities in non-polar environments.

## 4. Materials and Methods

### 4.1. Simulation Technique

#### 4.1.1. Polymer Chain Assembly Strategy

We developed comprehensive all-atom models for individual blocks and the complete block copolymers which take into account bond stretching, bond bending, and dihedral angle rotation around the equilibrium values as well as non-bonded van der Waals and electrostatic interactions. Individual monomer structures (Figure 1) were generated using standard molecular building tools. However, constructing polymer chains from these monomers presented a significant challenge due to their bulky side groups. Attempting to align monomers linearly to form a chain resulted in severe steric clashes that could not be resolved even through standard energy minimization algorithms. To overcome this, we developed a custom utility script employing a “step-wise assembly” protocol. First, monomers were placed sequentially along a central axis. Instead of positioning them at standard bond lengths, they were initially separated by a larger distance (5–7 Å). Then, each subsequent monomer was rotated around this axis by a fixed angle (e.g., 90°) to further minimize initial steric overlap. This pre-organized structure served as the input for an MD relaxation phase. The simulation began with a reduced integration time step and strong kinetic energy dissipation to gently guide the atoms into place. Once the system stabilized, the time step was increased to the standard value (*h* = 0.001 ps). During this process, bond lengths converged to the values defined by the force field, and the conformational properties of the chain relaxed to stationary states.

A similar protocol was employed to covalently link two distinct polymer blocks, facilitating the construction of the final block copolymer architectures.

#### 4.1.2. Solvation and System Preparation

To model isolated polymer molecules in heptane, we first prepared a condensed solvent phase using a standard equilibration procedure. Namely, heptane molecules were initially arranged on a simple cubic lattice with sufficient spacing to prevent atomic overlap. An MD simulation was performed while dynamically adjusting the simulation box dimensions to achieve the target experimental density. The relaxed polymer chain was then inserted into the center of the solvent box. Any solvent molecules overlapping sterically with the polymer were removed to eliminate high-energy contacts. The resulting configuration served as the starting point for final equilibration under an NPT ensemble until thermodynamic equilibrium was reached.

For the single-chain conformational study, four independent computational experiments were conducted for each polymer type (**PM_50_** and **PNP_50_**) under periodic boundary conditions, utilizing a simulation box populated with 1500 heptane molecules to mimic a dilute solution regime. These runs were evenly distributed across two distinct thermal conditions, 300 K and 350 K. The systems were initially equilibrated to their target density using an NPT ensemble before transitioning to production molecular dynamics runs lasting between 220 and 250 ns under an NVT ensemble. By maintaining polymer concentrations at low levels (5.1 wt% for **PM_50_** and 8.1 wt% for **PNP_50_**), we effectively isolated individual chains, preventing interactions with periodic images. All simulations originated from initial configurations resembling loose globules, generated through the partial relaxation of extended linear chains in a vacuum.

For block copolymer systems, simulations were conducted with varying numbers of chains (1, 2, and 4 molecules) to investigate concentration effects ranging from dilute (single chain) to semi-dilute (25 wt%) regimes. Specifically, we analyzed **PM_10_-*b*-PNP_40_** copolymers to understand the interplay between the distinct blocks during aggregation.

#### 4.1.3. Simulation Details and Computational Resources

All simulations were carried out using the AMBER99 force field [36,37] employing the PARM99 parameter set. This parameter set has been previously validated and demonstrated reliable performance in our earlier work [10]. Non-bonded van der Waals interactions were modeled using the conventional 12–6 Lennard-Jones (LJ) potential. Bond stretching, bond angle bending, and dihedral torsion potentials as well as electrostatic interactions between partial atomic charges were included. Partial atomic charges for the polymer atoms, which are not available in the standard AMBER library for these specific monomers, were assigned using the bond increment rules of the PCFF force field. A cutoff distance of 10.5 Å was applied to both LJ and Coulombic interactions. Periodic boundary conditions were employed in all three spatial dimensions (X, Y, and Z). Initial system equilibration was carried out at an elevated temperature of 600 K to ensure thorough mixing. During this stage, the simulation box size was gradually reduced until the equilibrium solution density was achieved. Further relaxation was performed in the NPT ensemble at temperatures of 300 and 350 K and atmospheric pressure until steady-state conditions were achieved. Subsequently, production simulations were performed in the NVT ensemble at T = 300 K and 350 K and atmospheric pressure. Temperature control was maintained using a collision-based thermostat [38] with a virtual particle mass of 1 amu and a collision frequency of 10 ps^−1^, while pressure was regulated via the Berendsen barostat [39].

The equations of motion were integrated with a time step of 0.001 ps. The total equilibration phase lasted approximately 50 ns, followed by a production run exceeding 150 ns. Equilibration and convergence to a steady state were verified by monitoring the time evolution of the system density, radius of gyration of single polymer molecules, and all contributions to the energy of the system.

Hydrogen bonds were identified based on standard geometric criteria. A bond was defined when both of the following conditions were met: (i) the donor–acceptor distance was less than 0.35 nm, and (ii) the hydrogen–acceptor distance was less than 0.25 nm.

Computations were carried out using the PUMA and PUMA-CUDA accelerated by NVIDIA CUDA graphics processor [40,41], specifically designed for the molecular dynamics simulation of polymers and biopolymers.

### 4.2. Experimental

#### 4.2.1. Materials and Characterization

Triethylamine (Et_3_N, Sigma), methacryloyl chloride (AVRA), aniline (Sigma), sodium chloride (NaCl, Merck), and 3,4,5-trimethoxybenzylamine (Sigma) were used as received. The detailed synthetic procedures for *N*-(3,4,5-trimethoxybenzyl)methacrylamide (**M**) and *N*-phenylmethacrylamide (**NP**) are explained in our previous work [10,42]. The 4-cyano-(dodecylsulfanylthiocarbonyl)sulfanylpentanoic acid (CDP), used as a chain transfer agent (CTA), was purchased from BLD Pharmatech Pvt. Ltd. (Bangalore, India). 2,2′-Azobis(isobutyronitrile) (AIBN, 98%) was purchased from SRL, recrystallized in methanol (MeOH), and used as an initiator during the RAFT polymerization reactions. The solvents used for ^1^H nuclear magnetic resonance (^1^H NMR) spectroscopy, such as DMSO-*d_6_* (99.9%, D) and CDCl_3_ (99.8%, D), were purchased from Cambridge Isotope Laboratories, Inc., USA. The commonly used organic solvents, including dichloromethane (DCM), hexane (mixture of isomers), heptane, ethyl acetate, diethyl ether, HPLC grade dimethylformamide (DMF), and tetrahydrofuran (THF), were acquired from Merck and used as received. The ^1^H NMR spectroscopic characterization of polymers was carried out on a JEOL ECS 400 MHz or Bruker Avance 500 MHz spectrometer at 25 °C. The molar mass and dispersity (*Đ*) of the polymers were determined by size exclusion chromatography (SEC) at 40 °C using DMF as the eluent, operated at a flow rate of 0.8 mL min^−1^. The SEC system consisted of a Waters 1515 HPLC pump, a Waters 2414 refractive index (RI) detector, one PolarGel-M guard column (50 × 7.5 mm), and two PolarGel-M analytical columns (300 × 7.5 mm). The instrument was calibrated with poly(methyl methacrylate) (PMMA) standards.

#### 4.2.2. Synthesis of a Homopolymer **PM_50_**, a Homopolymer **PNP_50_** and Block Copolymer (**PM_10_-*b*-PNP_40_**)

**Scheme 1:**
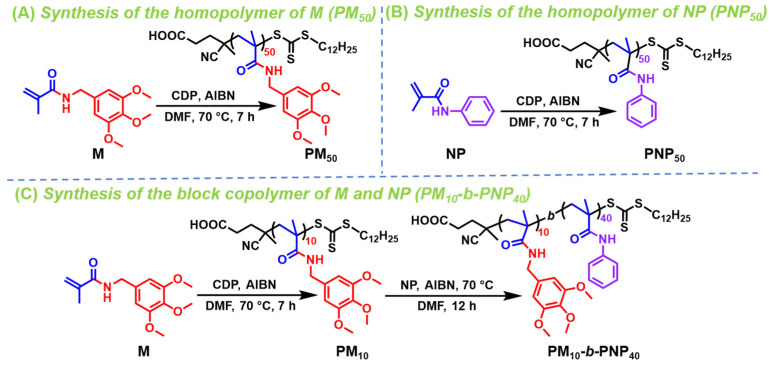
Synthesis strategy of homopolymers and block copolymer.

**Figure 14:**
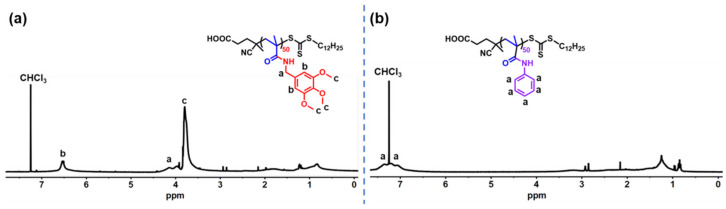
^1^H NMR spectra of (**a**) **PM_50_** and (**b**) **PNP_50_**.

**Figure 15:**
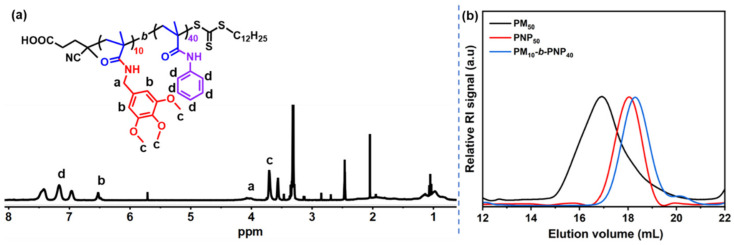
^1^H NMR spectrum of (**a**) **PM_10_-*b*-PNP_40_** and (**b**) SEC chromatogram of synthesized polymers.

To synthesize a gallol-functionalized homopolymer (**PM_50_**), **M** (1.0 g, 3.77 mmol), CDP (0.028 g, 0.07 mmol), and AIBN (2.29 mg, 0.014 mmol) were taken in DMF (3.5 mL) in a 20 mL septa-sealed glass vial (Scheme 1). Similarly, for the synthesis of the **NP** homopolymer, **NP** (1.0 g, 6.20 mmol), CDP (0.046 g, 0.115 mmol), and AIBN (3.78 mg, 0.023 mmol) were taken in DMF (3.5 mL) in another 20 mL septa-sealed glass vial. Both reaction mixtures were purged with N_2_ for 10 min and subsequently stirred in a polymerization block at 70 °C for 7 h. After completion of the polymerization, the vials were rapidly cooled in an ice-water bath and exposed to air. The resulting polymer solutions were precipitated into 60 mL of diethyl ether, affording sticky polymeric precipitates. The polymers were purified using standard procedures following previously reported methods [43,44]. The synthesized polymers were characterized by ^1^H NMR spectroscopy and SEC analysis (Figure 14 and Figure 15).

To synthesize block copolymer (**PM_10_-*b*-PNP_40_**), **PM_10_** was first synthesized in a similar way to **PM_50_**. Then, **PM_10_** (100 mg, 0.0377 mmol), **NP** (303.8 mg, 1.70 mmol), and AIBN (1.24 mg, 0.007 mmol) were taken in DMF (1.5 mL) in a 20 mL septa-sealed glass vial. After purging with N_2_ for 10 min, the reaction mixture was stirred at 70 °C in a polymerization block for 7 h. Upon completion of the polymerization, the reaction vials were rapidly cooled in an ice-water bath and exposed to air. The resulting polymer solution was precipitated into 60 mL of diethyl ether to afford a sticky polymeric material.

For purification, the precipitated polymer was dissolved in a minimum amount of acetone and reprecipitated into excess diethyl ether. This reprecipitation process was repeated four times, followed by vacuum drying to obtain the purified polymer. The synthesized polymers were characterized by ^1^H NMR spectroscopy and SEC analysis (Figure 15).

RAFT polymerization was performed using CDP as CTA and AIBN as the initiator in anhydrous DMF at 70 °C with a feed ratio of **M**:CDP = 54:1, to synthesize the gallol-pendant homopolymer (**PM_50_**, Scheme 1). The successful formation of **PM_50_** was confirmed by ^1^H NMR spectroscopy (Figure 14a), and SEC analysis revealed a number-average molecular weight (*M*_n,SEC_) of 13,200 g/mol (*Ð* = 1.28) (Figure 15b). Number-average molecular weight from ^1^H NMR spectroscopy (*M*_n,NMR_ = 13,660 g/mol) was calculated using the equation: *M*_n,NMR_ = (*DP*_n_ of **M** × molar mass of **M** + molar mass of CDP); where *DP*_n_ is the degree of polymerization, and *DP*_n_ value of 50 was estimated by comparing the terminal aliphatic -CH_2_(C*H*_2_)_9_CH_3_ protons of the CDP group (1.2–1.3 ppm) with those of the -OC*H*_3_ (3.8 ppm) protons of the gallol unit from **M**. The theoretical molecular weight obtained from gravimetric monomer conversion data was calculated using the following equation: *M*_n,theo_ = (([**M**]/[CDP]× molar mass of M × Conv.) + (molar mass of CDP)). The calculated theoretical molecular weight (*M*_n,theo_) for **PM_50_** was 13,400 g/mol. The close agreement among *M*_n,SEC_, *M*_n,NMR_ and *M*_n,theo_ values indicates good control over the RAFT polymerization process.

Similarly, RAFT polymerization of **NP** was carried out using CDP as the CTA and AIBN as the initiator in anhydrous DMF at 70 °C with a feed ratio of **NP**:CDP = 54:1 to afford the aniline-pendant homopolymer **PNP_50_** (Scheme 1). The structure of **PNP_50_** was confirmed by ^1^H NMR spectroscopy (Figure 14b). SEC analysis showed an *M*_n,SEC_ value of 9200 g/mol with a low *Ð* of 1.12 (Figure 15b). The molecular weight determined from ^1^H NMR spectroscopy (*M*_n,NMR_ = 8460 g/mol) was calculated using the equation: *M*_n,NMR_ = (*DP*_n_ of **NP** × molar mass of **NP** + molar mass of CDP). The *DP*_n_ value of 50 was determined by comparing the terminal aliphatic -CH_2_(C*H*_2_)_9_CH_3_ protons of the CDP end group (1.2–1.3 ppm) with the aromatic phenyl protons (7.2 ppm) of the **NP** repeating unit. Based on monomer conversion obtained by gravimetric analysis, the theoretical molecular weight was calculated using *M*_n,theo_ = (([**NP**]/[CDP]× molar mass of **NP** × Conv.) + (molar mass of CDP)). The theoretical molecular weight of **PNP_50_** was calculated to be 8600 g/mol, which showed good agreement with the experimentally determined values. For the synthesis of the block copolymer, **PM_10_** was employed as the macro-CTA, and chain extension with **NP** was carried out at a feed ratio of **PM_10_**:**NP** = 1:45 to obtain **PM_10_-*b*-PNP_40_** (Scheme 1). The structure of the block copolymer was confirmed by ^1^H NMR spectroscopy (Figure 15a). SEC analysis showed an *M*_n,SEC_ value of 8500 g/mol with a narrow *Ð* of 1.01 (Figure 15b). The number-average molecular weight determined from ^1^H NMR spectroscopy (*M*_n,NMR_ = 9100 g/mol) was calculated using the equation: *M*_n,NMR_ = (*DP*_n_ of **NP** × molecular weight of **NP**) + molecular weight of **PM_10_**. The *DP*_n_ value of 40 was estimated by comparing the gallol aromatic protons of **PM_10_** (6.9 ppm) with the aromatic phenyl protons of the **NP** unit (7.2 ppm). The theoretical molecular weight determined from monomer conversion data was calculated using: *M*_n,theo_ = ([**NP**]/[**PM_10_**] × molecular weight of **NP** × conversion) + molecular weight of **PM_10_**. The theoretical molecular weight of **PM_10_-*b*-PNP_40_** was determined to be 8900 g/mol. The close correspondence among *M*_n,SEC_, *M*_n,NMR_, and *M*_n,theo_ values further confirms the controlled nature of the RAFT polymerization. All molecular weight data are compiled in Table 3.

## 5. Conclusions

This atomistic MD study, supported by experimental validation, elucidates the conformational behavior and self-assembly of gallol-containing block copolymers in the non-polar solvent heptane. We demonstrate that intramolecular hydrogen bonding and dipolar interactions govern single-chain conformations, with **PNP_50_** forming stable globules and **PM_50_** exhibiting dynamic unfolding due to side-chain steric restrictions. At higher concentrations, **PNP**-driven intermolecular contacts promote percolation and aggregation, providing a mechanistic basis for the experimentally observed precipitation. These insights offer a molecular-level framework for tuning the self-organization of functional graft-like polymers in non-aqueous media.

## Figures and Tables

**Table 1:** Average number of van der Waals interchain contacts per chain in two- and four-chain systems.

System	PM–PM	PNP–PNP	PM–PNP	Intrachain
Two-chain (25 wt%)	307 ± 50	216 ± 50	36 ± 30	597 ± 80
Four-chain (25 wt%)	35 ± 30	320 ± 30	213 ± 30	782 ± 80

**Table 2:** Solvation shell analysis for **PM_10_-*b*-PNP_40_** systems at increasing concentration. Values represent averages ± standard deviation over the production trajectory.

System	Total Heptane per Chain	Heptane per Monomer	PM_10_ Block (per Monomer)	PNP_40_ Block (per Monomer)
Single-chain	55.9 ± 2.0	1.12 ± 0.04	1.95 ± 0.10	0.98 ± 0.05
Two-chain (25 wt%)	50.8 ± 0.7	1.02 ± 0.01	1.74 ± 0.05	0.90 ± 0.03
Four-chain (25 wt%)	41.1 ± 1.0	0.82 ± 0.02	1.41 ± 0.05	0.72 ± 0.02

**Table 3:** Summary of the results obtained from the synthesis of homo and block copolymers and their physiochemical characterizations.

Composition ^a^	Conv. ^b^ (%)	*M*_n,theo_ ^b^ (g/mol)	*M*_n,NMR_ ^a^ (g/mol)	*M*_n,SEC_ ^c^ (g/mol)	*Đ* ^c^
**PM_50_**	90	13,400	13,660	13,200	1.28
**PNP_50_**	92	8600	8460	9200	1.12
**PM_10_-*b*-PNP_40_**	86	8900	9100	8500	1.01

^a^ Determined by ^1^H NMR spectroscopy, ^b^ Calculated gravimetrically, ^c^ Obtained from the SEC.

## Data Availability

The original contributions presented in this study are included in the article. Further inquiries can be directed to the corresponding author.

## Abbreviations

The following abbreviations are used in this manuscript:

MD	Molecular Dynamics
**PM-*b*-PNP**	poly(gallol methacrylate)-*block*-poly(*N*-phenylmethacrylamide) copolymers
RAFT	Reversible addition fragmentation chain transfer
**NP**	*N*-phenylmethacrylamide
**M**	*N*-(3,4,5-trimethoxybenzyl)methacrylamide

## References

[1] Goswami P.K., Saikia P.J., Handique J.G. (2024). RAFT mediated dispersion copolymerization of glycidyl methacrylate and octadecyl methacrylate. J. Macromol. Sci. Part A Pure Appl. Chem..

[2] Shit S., Nayek S., Senapati D., De P. (2026). Design and synthesis of pH-sive polymers with dipeptide side-chain pendants for supramolecular self-assembly. J. Macromol. Sci. Part A Pure Appl. Chem..

[3] Kim H.J., Ishizuka F., Kuchel R.P., Chatani S., Niino H., Zetterlund P.B. (2024). RAFT dispersion PISA with poly(methyl methacrylate) as stabilizer block in alcohol/water: Unconventional PISA morphology transitions. Biomacromolecules.

[4] Penfold N.J.W., Yeow J., Boyer C., Armes S.P. (2019). Emerging trends in polymerization-induced self-assembly. ACS Macro Lett..

[5] Kang Y., Pitto-Barry A., Maitland A., O’Reilly R.K. (2015). RAFT dispersion polymerization: A method to tune the morphology of thymine-containing self-assemblies. Polym. Chem..

[6] Rieger J. (2015). Guidelines for the synthesis of block copolymer particles of various morphologies by RAFT dispersion polymerization. Macromol. Rapid Commun..

[7] Sahoo S., Gordievskaya Y.D., Bauri K., Gavrilov A.A., Kramarenko E.Y., De P. (2022). Polymerization-induced self-assembly (PISA) generated cholesterol-based block copolymer nano-objects in a nonpolar solvent: Combined experimental and simulation study. Macromolecules.

[8] Cao J., Li Y., Tan Y., Zhang L., Tan J. (2024). From RAFT emulsion polymerization to RAFT dispersion polymerization: A facile approach to tuning dispersities and behaviors of self-assembled block copolymers. Polym. Chem..

[9] Chowdhury P., Bauri K., De P. (2026). Pyroglutamate pendant block copolymers: Evolution of inverse and conventional morphologies from RAFT-mediated PISA. Chem. Sci..

[10] Shit S., Poddar P., Kurbatov A.O., Balabaev N.K., Chistyakov A.P., Kuznetsova E.V., Kramarenko E.Y., Chvalun S.N., De P. (2026). RAFT dispersion polymerization derived polymeric antioxidant nano-architectures: Morphology elucidated by molecular dynamics simulations. Eur. Polym. J..

[11] Acik G., Altinkok C. (2025). Biopolymers containing bile acid derivatives: Recent advances, material comparisons, and application prospects. J. Macromol. Sci. Part A Pure Appl. Chem..

[12] Su Y., Wang Y., Yang L., Min M., Yang J., Guo J., Zhu Y., Liu K. (2025). Effect of polyphenol intake and assisted reproductive outcomes in the female population: A systematic review. Food Funct..

[13] Kim S., Saha B., Boykin J., Chung H. (2022). Gallol containing adhesive polymers. J. Macromol. Sci. Part A Pure Appl. Chem..

[14] Mai V.C., Li D., Duan H. (2023). Phenolic-compound-based functional coatings: Versatile surface chemistry and biomedical applications. Langmuir.

[15] Fan G., Cottet J., Rodriguez-Otero M.R., Wasuwanich P., Furst A.L. (2022). Metal-phenolic networks as versatile coating materials for biomedical applications. ACS Appl. Bio Mater..

[16] Xu C., Huang R., Yu M., Zhang S., Wang Y., Chen X., Hu Z., Wang Y., Xing X. (2024). Facile bond exchanging strategy for engineering wet adhesion and antioxidant/antibacterial thin layer over a dynamic hydrogel via the carbon dots derived from tannic acid/*ε*-polylysine. ACS Appl. Mater. Interfaces.

[17] Shao Q., Jiang S. (2015). Molecular understanding and design of zwitterionic materials. Adv. Mater..

[18] Asha A.B., Chen Y., Narain R. (2021). Bioinspired dopamine and zwitterionic polymers for non-fouling surface engineering. Chem. Soc. Rev..

[19] Chang Y. (2022). Designs of zwitterionic polymers. J. Polym. Res..

[20] Budkov Y.A., Brandyshev P.E., Kalikin N.N. (2023). Theory of self-coacervation in semi-dilute and concentrated zwitterionic polymer solutions. Soft Matter.

[21] Kumar R., Li W., Sumpter B.G., Muthukumar M. (2019). Understanding the effects of dipolar interactions on the thermodynamics of diblock copolymer melts. J. Chem. Phys..

[22] Glova A., Larin S., Falkovich S., Nazarychev V., Tolmachev D., Lukasheva N., Lyulin S. (2017). Molecular dynamics simulations of oligoester brushes: The origin of unusual conformations. Soft Matter.

[23] Birshtein T., Polotsky A., Glova A., Amoskov V., Mercurieva A., Nazarychev V., Lyulin S. (2018). How to fold back grafted chains in dipolar brushes. Polymer.

[24] Glova A.D., Larin S.V., Nazarychev V.M., Karttunen M., Lyulin S.V. (2020). Grafted dipolar chains: Dipoles and restricted freedom lead to unexpected hairpins. Macromolecules.

[25] Budkov Y.A., Kalikin N.N., Kolesnikov A.L. (2021). Molecular theory of the electrostatic collapse of dipolar polymer gels. Chem. Commun..

[26] Gordievskaya Y.D., Budkov Y.A., Kramarenko E.Y. (2018). An interplay of electrostatic and excluded volume interactions in conformational behavior of a dipolar chain: Theory and computer simulations. Soft Matter.

[27] Verduzco R., Li X., Pesek S.L., Stein G.E. (2015). Structure, function, self-assembly, and applications of bottlebrush copolymers. Chem. Soc. Rev..

[28] Fei H.F., Yavitt B., Hu X., Kopanati G., Ribbe A., Watkins J. (2019). Influence of molecular architecture and chain flexibility on the phase map of polystyrene-*block*-poly(dimethylsiloxane) brush block copolymers. Macromolecules.

[29] Zamurovic M., Christodoulou S., Vazaios A., Iatrou E., Pitsikalis M., Hadjichristidis N. (2007). Micellization behavior of complex comblike block copolymer architectures. Macromolecules.

[30] Zhulina E., Sheiko S., Borisov O.V. (2022). Theoretical advances on molecular bottlebrushes and comblike (co)polymers: Solutions, gels, and self-assembly. Soft Matter.

[31] Dalsin S., Rions-Maehren T., Beam M., Bates F., Hillmyer M., Matsen M. (2015). Bottlebrush block polymers: Quantitative theory and experiments. ACS Nano.

[32] Lebedeva I., Zhulina E., Borisov O. (2023). Polymorphism of self-assembled colloidal nanostructures of comblike and bottlebrush block copolymers. Colloid Polym. Sci..

[33] Gordievskaya Y.D., Kramarenko E.Y. (2019). Conformational behavior of a semiflexible dipolar chain with a variable relative size of charged groups via molecular dynamics simulations. Soft Matter.

[34] Gordievskaya Y.D., Kramarenko E.Y. (2021). Conformational transitions and helical structures of a dipolar chain in external electric fields. Soft Matter.

[35] Merzliakova T.Y., Gordievskaya Y.D., Kramarenko E.Y. (2023). Conformational behavior of a single dipolar chain under stretching force. Macromolecules.

[36] Weiner S.J., Kollman P.A., Case D.A., Singh U.C., Ghio C., Alagona G., Profeta S., Weiner P. (1984). A new force field for molecular mechanical simulation of nucleic acids and proteins. J. Am. Chem. Soc..

[37] Wang J., Wolf R.M., Caldwell J.W., Kollman P.A., Case D.A. (2004). Development and testing of a general Amber force field. J. Comput. Chem..

[38] Lemak A.S., Balabaev N.K. (1995). A comparison between collisional dynamics and Brownian dynamics. Mol. Simul..

[39] Berendsen H.J.C., Postma J.P.M., Van Gunsteren W.F., Di Nola A., Haak J.R. (1984). Molecular dynamics with coupling to an external bath. J. Chem. Phys..

[40] Glyakina A.V., Balabaev N.K., Galzitskaya O.V. (2009). Mechanical unfolding of proteins L and G with constant force: Similarities and differences. J. Chem. Phys..

[41] Lyulin A.V., Balabaev N.K., Michels M.A.J. (2002). Correlated segmental dynamics in amorphous atactic polystyrene: A molecular dynamics simulation study. Macromolecules.

[42] Mondal A., Pal A., Sarkar S., Datta R., De P. (2024). Antioxidant polymers with phenolic pendants for the mitigation of cellular oxidative stress. Biomacromolecules.

[43] Karmakar S., Dinda P., Barik S., Das J., Basak A., Mandal T.K. (2025). RAFT-synthesized citrulline-derived polymethacrylates with tunable UCST for dye encapsulation and a fluorescent FITC-tagged LCST conjugate. ACS Appl. Polym. Mater..

[44] Schaefer S., Melodia D., Pracey C., Corrigan N., Lenardon M.D., Boyer C. (2024). Mimicking charged host-defense peptides to tune the antifungal activity and biocompatibility of amphiphilic polymers. Biomacromolecules.

